# Patient and researcher experiences of patient engagement in primary care health care research: A participatory qualitative study

**DOI:** 10.1111/hex.13542

**Published:** 2022-07-22

**Authors:** Sophie Béland, Mireille Lambert, Alannah Delahunty‐Pike, Dana Howse, Charlotte Schwarz, Maud‐Christine Chouinard, Kris Aubrey‐Bassler, Fred Burge, Shelley Doucet, Alya Danish, Olivier Dumont‐Samson, Mathieu Bisson, Alison Luke, Marilyn Macdonald, André Gaudreau, Judy Porter, Donna Rubenstein, Véronique Sabourin, Cathy Scott, Mike Warren, Linda Wilhelm, Catherine Hudon

**Affiliations:** ^1^ Département de Médecine de Famille et de Médecine D'urgence Université de Sherbrooke Sherbrooke Quebec Canada; ^2^ Centre Intégré Universitaire de Santé et de Services Sociaux du Saguenay‐Lac‐Saint‐Jean Chicoutimi Quebec Canada; ^3^ Department of Family Medicine Dalhousie University Halifax Nova Scotia Canada; ^4^ Primary Healthcare Research Unit, Faculty of Medicine Memorial University St‐John's Newfoundland and Labrador Canada; ^5^ Department of Nursing and Health Sciences University of New Brunswick St‐John's Newfoundland and Labrador Canada; ^6^ Faculté des Sciences Infirmières Université de Montréal Montréal Quebec Canada; ^7^ Canadian Institutes of Health Research Ottawa Ontario Canada; ^8^ School of Nursing, Faculty of Health Dalhousie University Halifax Nova Scotia Canada; ^9^ Patient Partner; ^10^ Centre de Recherche du Centre Hospitalier Universitaire de Sherbrooke Centre Hospitalier Universitaire de Sherbrooke Sherbrooke Quebec Canada

**Keywords:** participatory action research, patient engagement, patient‐oriented research

## Abstract

**Background:**

Studies have highlighted common challenges and barriers to patient engagement in research, but most were based on patient partners' or academic researchers' experiences. A better understanding of how both groups differentially experience their partnership could help identify strategies to improve collaboration in patient engagement research.

**Aim:**

This study aimed to describe and compare patient partners' and academic researchers' experiences in patient engagement research.

**Methods:**

Based on a participatory approach, a descriptive qualitative study was conducted with patient partners and academic researchers who are involved in the PriCARE research programme in primary health care to examine their experience of patient engagement. Individual semi‐structured interviews with patient partners (*n* = 7) and academic researchers (*n* = 15) were conducted. Academic researchers' interview verbatims, deidentified patient partners' summaries of their interviews and summaries of meetings with patient partners were analysed using inductive thematic analysis in collaboration with patient partners.

**Results:**

Patient partners and academic researchers' experiences with patient engagement are captured within four themes: (1) evolving relationships; (2) creating an environment that fosters patient engagement; (3) striking a balance; and (4) impact and value of patient engagement. Evolving relationships refers to how partnerships grew and improved over time with an acceptance of tensions and willingness to move beyond them, two‐way communication and leadership of key team members. Creating an environment that fosters patient engagement requires appropriate structural support, such as clear descriptions of patient partner roles; adequate training for all team members; institutional guidance on patient engagement; regular and appropriate translation services; and financial assistance. For patient partners and academic researchers, striking a balance referred to the challenge of reconciling patient partners' interests and established research practices. Finally, both groups recognized the value and positive impact of patient engagement in the programme in terms of improving the relevance of research and the applicability of results. While patient partners and academic researchers identified similar challenges and strategies, their experiences of patient engagement differed according to their own backgrounds, motives and expectations.

**Conclusion:**

Both patient partners and academic researchers highlighted the importance of finding a balance between providing structure or guidelines for patient engagement, while allowing for flexibility along the way.

**Patient or Public Contribution:**

Patient partners from the PriCARE research programme were involved in the following aspects of the current study: (1) development of the research objectives; (2) planning of the research design; (3) development and validation of data collection tools (i.e., interview guides); (4) production of data (i.e., acted as interviewees); (5) validation of data analysis tools (code book); (6) analysis of qualitative data; and (7) drafting of the manuscript and contributing to other knowledge translation activities, such as conference presentations and the creation of a short animated video.

## 1 INTRODUCTION

Patient engagement in health research has received growing interest within academic literature and policy discourse in recent years.^1^, ^2^, ^3^, ^4^ Defined as ‘meaningful and active collaboration of patients in the governance, priority setting, execution and translation of research’,^5^ this approach aims to shift the paradigm of academic researchers assuming the role of ‘expert’ to one where power and responsibly are shared between patients and academic researchers, and research is coconstructed to reach common goals.^6^, ^7^ Patient engagement can produce research that is more closely aligned with the needs and realities of those who will be directly impacted by its application. Increased recognition of the value of patient engagement has led governments and funding agencies to promote and even mandate the use of this approach in research.^5^, ^8^

The growing interest for patient engagement in health research is reflected by the high number of publications on topics related to this approach. Knowledge gained from this literature has led to the elaboration of strategies and best practice recommendations for patient engagement.^9^, ^10^, ^11^, ^12^, ^13^, ^14^, ^15^, ^16^, ^17^ In addition, several reviews have begun to map the benefits of patient engagement^2^, ^4^, ^18^, ^19^, ^20^ that affect all stages of research, including producing more relevant research topics; improved participant recruitment and retention; creation of data collection tools better suited to target populations; and enriched interpretations of study results.^2^, ^19^

Although the literature supports the added value of patient engagement, and academic researchers initially seem willing to involve patients in research, their attitudes towards applying this approach concretely in the context of research remain mixed, ranging from resistant, ambivalent to positive.^21^, ^22^, ^23^ This suggests that certain factors may deter some teams from using this approach. Indeed, multiple qualitative studies have highlighted common challenges and barriers to patient engagement, such as power dynamics, heavy time commitment, resources required, differences in knowledge and expectations between patient partners and academic researchers and difficulty recruiting patient partners.^24^, ^25^, ^26^, ^27^, ^28^, ^29^ To date, most studies have reported on experiences of patient engagement from the perspective of either patient partners or academic researchers, but rarely from both groups simultaneously. There is a need to explore and compare patient partners' and academic researchers' lived experiences and challenges with patient engagement.^30^ This would help identify aspects that matter most to each party, including perceived challenges and areas that would be most helpful to address.

One example of patient engagement in health research is the PriCARE research programme, a multiple‐case embedded mixed‐methods study design conducted since 2018 in five Canadian provinces: New Brunswick, Newfoundland‐and‐Labrador, Nova Scotia, Quebec and Saskatchewan.^31^ The aim is to study the implementation of a case management (CM) intervention for frequent users of health care services with chronic diseases and complex care needs in primary care clinics across Canada. The objectives are to identify the facilitators and barriers of CM implementation in primary care clinics across Canada, to explain and understand the relationships between the actors, contextual factors, mechanisms and outcomes of the CM intervention and to identify the next steps towards CM spread in primary care across Canada. In each province, one or two primary care clinics were recruited to implement and evaluate the CM intervention, in partnership with patient partners. The intervention, led by primary care nurses, focuses on four components: (1) patient evaluation; (2) individualized care plan; (3) care coordination; and (4) self‐management. The PriCARE outcomes are knowledge of the facilitators and barriers to CM implementation in different primary care contexts and jurisdictions; an evidence‐based CM intervention, adapted to different provincial contexts; explanations about how CM works; and recommendations on future steps for scalability of CM in primary care across Canada. The PriCARE programme is detailed elsewhere.^32^

Better understanding how both patient partners and academic researchers differentially experience their partnership could help inform research teams', funders' and policy‐makers' future efforts to engage patients in research by highlighting potential strategies that can improve communication and collaboration. The aim of this study is to describe and compare experiences of patient engagement among patient partners and academic researchers involved in the PriCARE research programme.

## 2 MATERIALS AND METHODS

### 2.1 Patient engagement in the PriCARE research programme

In each of the five provinces participating in the PriCARE research programme, one to two patient partners, that is, patients or family caregivers of patients with experience of complex health care needs and of the health care system, were recruited to work closely with the local academic research team. All patient partners received a 1‐h training on patient engagement in research developed by the Patient Partners Initiative (Université de Sherbrooke). The roles of patient partners included review of data collection tools, cofacilitation of the training of case managers, advising on participant recruitment, programme monitoring, data analysis and interpretation, codevelopment, delivery of conference presentations and contributing to publications. Along with academic research team members, patient partners took part in the programme's governance structure as members of the steering committee and contributed to the planning and execution of the research programme. Patient partners were remunerated for their engagement and reimbursed for activities in the context of the PriCARE research programme. In addition, the academic research team recognized patient partners' involvement in the PriCARE research programme by publicly acknowledging their contribution to programme outcomes (e.g., publication authorship and tool development).

### 2.2 Conceptual model

The PriCARE research programme is conducted in accordance with Canada's Strategy for Patient‐Oriented Research (SPOR)—Patient Engagement Framework.^5^ The Patient Engagement Framework outlines a set of key principles to be adopted by stakeholders collaborating in patient‐oriented research, such as patients, academic researchers, decision‐makers, health organizations, provincial/territorial health authorities, academic institutions, charities and the pharmaceutical sector, to guide and optimize patient engagement. These principles include promoting the inclusivity and diversity of patient partners, providing adequate support to patient partners, ensuring mutual respect among stakeholders and encouraging cobuilding and active collaboration.

### 2.3 Study design

To fulfil the current study aim, a descriptive qualitative study design^33^, ^34^ was used to provide a rich description of the team members' experiences of patient engagement in the PriCARE research programme. This study was conducted using a participatory approach,^35^ in which academic researchers and patient partners actively collaborated and contributed to all stages of the research process. Participatory research may be defined as a ‘systematic inquiry, with the collaboration of those affected by the issue being studied, for purposes of education and taking action or effecting change’.^36^ In this study, patient partners and academic researchers were involved in the following stages of the research process: identifying the research question and study objectives; development and validation of study tools; participation in interviews facilitated by external research interviewers; data analysis and interpretation; and dissemination of results. Further, in line with the participatory approach, team members acted both as participants from whom the qualitative data were obtained, and as members of the team involved in the data analysis. This decision was made considering the participatory approach design, which we see as a strength. The role of team members in data collection and analysis is detailed in the following sections.

### 2.4 Participants and recruitment

All members of the steering committee (*n* = 25) were invited by email to participate. Seven patient partners and fifteen academic research team members, including seven research assistants, five principal investigators, two coinvestigators and one postdoctoral fellow, agreed to participate, from four out of the five participating Canadian provinces.

### 2.5 Data collection

Individual semi‐structured interviews were conducted by two interviewers with expertise in qualitative research, one programme manager referred by a patient partner and one doctoral student, both external to the research team. The interview guide was generated by a postdoctoral student in collaboration with patient partners, and produced in both English and French, following recommendations of the SPOR Framework described above. The guide was then reviewed and validated by all members of the research team. The interview guide included open‐ended questions on perceptions of (1) the contribution of patient partners; (2) resources allocated to support patient engagement; (3) events or incidents that occurred during the process; (4) prior expectations regarding patient engagement; and (5) expected outcomes of patient engagement.

Interviews were conducted virtually in the mother tongue of the interviewee (English or French). All interviews were conducted between December 2020 and February 2021, about 2 years into the PriCARE research programme. Research interviews were digitally recorded and transcribed verbatim. To maintain confidentiality, deidentified summaries of patient partner interviews were produced by two interviewers. A validation meeting then occurred, where patient partners reviewed and approved these summaries.

### 2.6 Analysis

Qualitative data were analysed using an inductive thematic analysis approach.^37^ First, three research assistants of the PriCARE programme (A. D. P., D. H. and C. S.), who were also interviewed in this study, developed a preliminary code book based on topics identified during an initial reading of three transcripts from academic team member interviews. With the guidance of the two external interviewers, patient partners reviewed their interview summaries and generated an additional list of codes, which were incorporated into the code book and validated. Second, the code book was used by five research assistants (A. D. P., D. H., C. S., O. D. S. and M. L.) to code the transcripts and patient partner interview summaries using NVIVO server software (QSR International Pty). All transcripts and summaries were coded by at least two coders and the coding team met multiple times to discuss and refine their coding approach. Coded data were entered into a table, organized by theme and by participant type (academic researcher or patient partner) so that similarities and differences between the two groups could be captured. During a 1‐h virtual team meeting, patient partners reviewed and commented on this table, shaping how the data were interpreted and which themes were more relevant. Patient partners' comments and feedback were noted and integrated in the table. Finally, a research assistant external to the research team (S. B.), and not involved in PriCARE, reviewed the table and identified four key themes. These key themes were reviewed and validated by all authors. The trustworthiness of the analysis was enhanced through researchers' triangulation and team validation.

### 2.7 Ethical considerations

This study was reviewed and approved by Ethics Review Boards in each of the four participating provinces: Comité d'éthique du Centre intégré universitaire de santé et services sociaux (CIUSSS) de l'Estrie‐ CHUS; Research Ethics Boards Horizon Health Network; University of New Brunswick Research Ethics Board, Research Ethics Board Memorial University; and Nova Scotia Health Research Ethics Board. All participants provided informed consent to participate in the study.

## 3 RESULTS

Table 1 presents participants' sociodemographic characteristics. A total of 22 participants (72.7% female), including 7 patient partners and 15 academic research team members, were interviewed over 30–60 min. Most participants were between 35 and 44 years of age, spoke English, had patient engagement training in research and had previous patient engagement experience in research.

**Table 1:** Sociodemographic characteristics of the participants (*N* = 22).

	Academic researcher (*n* = 15)	Patient partner (*n* = 7)
	*n* (%)	*n* (%)
Gender		
Female	11 (73.3)	5 (71.4)
Age		
25–34 years old	2 (13.3)	0 (0.0)
35–44 years old	7 (46.7)	0 (0.0)
45–54 years old	4 (26.7)	2 (28.6)
55–64 years old	0 (0.0)	4 (57.1)
≥65 years old	2 (13.3)	1 (14.3)
Location		
Newfoundland‐and‐Labrador	2 (13.3)	1 (14.3)
New Brunswick	4 (26.7)	2 (28.6)
Nova Scotia	3 (20.0)	2 (28.6)
Quebec	6 (40.0)	2 (28.6)
First language		
English	10 (66.7)	5 (71.4)
French	5 (33.3)	2 (28.6)
Time of involvement in PriCARE		
Upon grant submission	6 (40.0)	2 (28.6)
Initial implementation	5 (33.3)	0 (0)
During the implementation	3 (20.0)	5 (71.4)
Recently	1 (6.7)	0 (0.0)
Had patient engagement training in research	8 (53.3)	4 (57.1)
Previous patient engagement experience in research	10 (66.7)	5 (71.4)
	Mean (SD)	Mean (SD)
Years of PE experience in research^a^	5.8 (2.6)	10.5 (8.4)

Abbreviation: SD, standard deviation.

^a^ Only for participants who had previous patient engagement experience in research (academic researcher *n* = 5; patient partner *n* = 4).

Findings are presented below according to four overarching themes that best characterize members' experiences: (1) evolving relationships; (2) creating an environment that fosters patient engagement; (3) striking a balance; and (4) impact and value of patient engagement. Table 2 presents the themes, subthemes and exemplary quotes emerging from the analysis. Table 3 presents a running example of patient engagement experience in PriCARE. This example is used across the description of the four overarching themes.

**Table 2:** Overarching themes, subthemes and quotes that best characterize patient partners' and research team members' experiences.

Theme	Subtheme	Exemplary quotes
Evolving relationships	Experiencing tension	*Another topic that was discussed amongst most patient partners was how comfortable they have been to participate in this programme. Patient partners mentioned that other research team members have been very responsive to their questions and/or concerns, but it has taken some time to get this place. They highlighted that the research team has come a long way since the beginning*. (Summary of patient partner interviews)
	Two‐way communication	*They had their own meetings that were separate from the research coordinators. And in those meetings, they were… given that opportunity to provide complete feedback on how things could be improved with the research team or if there were any other needs that they would have liked to have seen better addressed. And then that feedback in its entirety would be reported back to the research team members, the PIs. So that communication channel was open*. (Researcher interview #14)
	Leadership of key team members	*They voiced their concern that at times it felt like patient partner views and recommendations were not taken seriously and/or with the same consideration as academic/research views (e.g., the initial clinic patient questionnaire). This was overcome by strong leadership by the Nominated PI (NPI) and other inclusive and supportive research team members*. (Summary of the validation meeting with patient partners)
Creating an environment that fosters patient engagement	Expectations	*Generally, a majority of the patient partners discussed how there could have been more time up front dedicated to discussing expectations for all research team members, which includes the patient partners. A patient partner suggested that there could have been more regular check‐ins as the programme progressed, to ensure effective communication and role clarification. Some patient partners also recommended revisiting the Terms of Reference and/or expectations moving forward*. (Summary of patient partner interviews)
	Training	*Patient partners have come and gone throughout the life of the programme and though it is understandable as to why, it can create a challenge to bring new patient partners up to speed on the work‐to‐date. On the flipside, newer patient partners also mentioned how it can be hard to catch up with what is going on. Some patient partners felt that they did not receive enough training or information to feel up to speed. Another patient partner mentioned that sharing lots of new documents, such as papers, processes, and maps, can be extremely overwhelming and might not the be best way to orient new patient partners*. (Summary of the validation meeting with patient partners)
	Institutional guidance	*But, you know, we're held up trying to get that person in place because we need to establish what their role will look like. And that depends on how we can pay them, and all that kind of thing. So that's my little vent on that, and frustration. (…) It's been a frustration for me. But what it really reflects is a disconnect between, you know, this discourse that's out there nationally from the major funding agencies about engaging patients meaningfully, and what that looks like in terms of the time we request of them, the work we request of them ‐ which is not insignificant. We need to be able to pay them appropriately and accordingly. And there's a lot of variation across the provinces. And I think perhaps for whatever reason, I've met the most resistance or difficulty here at [the University]*. (Researcher interview #5)
	Financial support	*It's been basically a year trying to sort through the bureaucracy here around the existing remuneration arrangements for patient partners (…). You know, the national lead, and the PIs were all, you know, very well intentioned, wanting to remunerate the patient partners appropriately and generously, according to some of the other parameters that are out there. But then what we're faced with is actually seeing that through and working with the infrastructure that's available to me. (Researcher interview #5)*
	Language barriers	*There is also the fact that English is not our first language and we have to lead meetings in English, we have to create relationships in English, so we have to be interested in the person, what he lives, what he does, his activities, his hobbies, so you know, we have to generate a conversation, so it's not easy either. So I think that this plays a little bit on the commitment of the patients, yes because it plays on our relationship with the patients, when it's in French, it's easy, we know how to create the relationship, when it's in English, we're searching for the right words and it's more difficult to create a good relationship, so it's more difficult to engage the patient in the programme*. (Researcher interview #1)
Striking a balance	Patient perspective	*And if you're going to do the really deep listening about the patient partner side of that, how do you know at the right moment when you need to also ensure they understand your voice in that as well without it coming across in a hierarchical way or I'm the researcher, this is why we do this?* (Researcher interview #10)
	Time commitment	*A patient partner mentioned that sometimes there are too many emails and too much paperwork to review. This patient partner appreciated that the other research team members were asking for their perspective, but all of the emails and paperwork felt overwhelming at times*. (Summary of patient partner interviews)
Impact and value of patient engagement	Diverse perspectives and innovative ways of doing research	*Well, I think that it [patient perspective] counterbalances our vision as academic researchers where, as I said, we are in the validity, the scientificness in the things that we repeat in the same way, always the same, always the same, so the patients force us to get out of that paradigm, of the scientific effect. That's when we realize that even if we get out of that paradigm, our data are still valid, sometimes even more so, and that we can do good research with good outcomes, and I think that's the first thing that the patient does, he takes us out of our comfort zone*. (Researchers interview #1)
	Improved research relevance and applicability	*Sometimes when we're doing research, you know, we have our protocol and we say we're going to do this and we're going to follow it through. But sometimes, too, it's… I find the patient partners help us to take a step back and say, okay, but does it really make sense to do this? And if we don't do this, is it the end of the world? And typically, not. You know, the project is still going to give us meaningful information. So just sometimes… Not to say to take the rigour out of the research, but it just reminds us of at the end of the day, what is it we're hoping to achieve, and being flexible and adaptable when changes need to be made, particularly, you know, when it makes sense to make changes from a patient lens*. (Researchers interview #12)

**Table 3:** Development of patient‐oriented guidelines for administering the patient questionnaires, an example of patient engagement experience in PriCARE.

Before data collection, patient partners raised concerns regarding the administering of patient questionnaires, and with specific questionnaire items. Their concerns included lack of information when introducing questionnaires to patients; unclear terms, phrases or questions; inappropriate questions for patients with multiple health conditions; and issues with sociodemographic questions, such as lack of consideration for gender diversity. The main challenge was reconciling patients' concerns with the academic researchers' requirements to maintain questionnaire validity. Nevertheless, academic researchers and patient partners decided to work together to address this challenge. Team members engaged in a participatory process to discuss these concerns, review the questionnaires and identify solutions. This process resulted in the development of patient‐oriented guidelines for administration of the questionnaires.^38^

### 3.1 Evolving relationships

One aspect that seemed to unite both academic research team members and patient partners in their experience of patient engagement was the feeling that relationships evolved throughout the life of the programme. The partnership grew and improved over time based on an acceptance of tension and willingness to move past it, two‐way communication, willingness to collaboratively problem solve and leadership of key team members.

#### 3.1.1 Experiencing tension

Patient partners reported initially feeling that they were not heard or taken seriously as research collaborators, and academic research team members observed this discomfort. Despite initial challenges in relationship building, patient partners also described a sense of growing comfort in expressing their opinions and of feeling increasingly involved in decision‐making. One patient partner recounted how, over time, he developed the habit of preparing talking points and sharing them during meetings.

#### 3.1.2 Two‐way communication

Maintaining regular contact between team members was identified by both groups as a factor that contributed to relationship growth. As the programme evolved, researchers and patient partners decided to provide dedicated time and space to exchange with team members, and this significantly contributed to developing a sense of trust within the team. More specifically, periodic patient partner‐only ‘check‐in’ meetings were highlighted as particularly conducive to developing connections, facilitated peer‐to‐peer support and provided a space for patient partners to voice concerns. Participants also highlighted the development of patient‐oriented guidelines for administering patient questionnaires as a turning point in their working relationship (Table 3). Patient partners felt that their concerns were being heard and addressed, and academic researchers found that patient partners understood the importance of providing scientifically valid questionnaires.

#### 3.1.3 Leadership of key team members

Participants felt that the interpersonal abilities and open‐mindedness of local team leaders made patient partners feel heard, supported and valued throughout the research process, which contributed to their growing comfort as members of the team. In addition, team responsiveness to feedback contributed to the success of working relationships. As an example, after patient partners voiced their concerns of not being adequately included in planning and decision‐making, their concerns were quickly addressed, and changes to team functioning were made accordingly.

### 3.2 Creating an environment that fosters patient engagement

Among both groups, it was unanimous that appropriate structural support for patient engagement was needed. Clear descriptions of patient partner and academic researcher roles and expectations; adequate training for all team members; regular and appropriate language translation services; institutional guidance on patient engagement; and financial remuneration were key elements to support patient partner involvement in research.

#### 3.2.1 Expectations

Discrepancies and uncertainties regarding the roles of patient partners and the degree and nature of patient engagement in the programme contributed to initial discomfort within the team. Some academic team members were not expecting patient partners to be as actively engaged as they became. Patient partners' understanding of their roles varied, with some voicing role uncertainty and lack of clear guidelines, while others had clear expectations of being actively involved in the planning and implementation of the programme. Lack of standardization of patient engagement across study sites and personnel turnover contributed to these misunderstandings. Ultimately, this left some academic research team members uncertain of how to integrate patient partners in the research process, resulting in infrequent communication with patient partners, or even exclusion of patient partners from some activities. For patient partners, this contributed to feelings of frustration and ‘disrespect’.

#### 3.2.2 Training

Lack of training and prior experience with patient engagement were also noted by both groups as factors that could affect the development of relationships in the patient engagement process. For example, patient partners without training or previous experience felt intimidated and hesitant to speak in meetings, whereas more experienced patient partners spoke freely, potentially creating power imbalances within the team.

#### 3.2.3 Language barriers

Interviews revealed two types of language barriers that had to be addressed to facilitate patient engagement. First, both groups agreed that the use of scientific terms and jargon reduced the potential for patient partner contribution. For example, during the process of developing patient‐oriented guidelines (Table 3), patient partners recognized that validated questionnaires could not be modified, but proposed to provide additional clarifications to questions that were more difficult to understand. In addition, patient partners noted how their lack of knowledge of the correct ‘lingo’ resulted in some contributing very little at meetings. Second, strategies were devised to overcome the fact that some team members were unilingual (English or French), such as translation of research materials and real‐time interpretation of meeting discussions. This was necessary to ensure fruitful interactions and discussions among all team members.

#### 3.2.4 Institutional guidance

To facilitate patient engagement, both groups recognized the need for stronger guidance from funding agencies on how to engage patient partners in research, for both the academic researchers and the institutions in which they conduct the research. Indeed, academic researchers highlighted a disconnect between how patient engagement is currently encouraged and, in some cases mandated by funding agencies, and how patient engagement is actually supported in practice. For example, some academic researchers were unsure of how to manage patient compensation as no clear guidance from their institution existed to support this process.

#### 3.2.5 Financial support

Both groups agreed that patient partner remuneration and financial support for travel or meeting expenses were essential to support patient engagement. However, misunderstandings and challenges with patient partner remuneration occurred. Both groups expressed a perceived imbalance between the amount of work put in and remuneration received. For example, some patient partners mentioned being compensated for attending meetings, but not necessarily for the time spent to prepare for them.

### 3.3 Striking a balance

Academic researchers and patient partners discussed the notion of ‘striking a balance’ on many aspects over the course of the programme. They expressed difficulty determining to what extent the research programme could integrate the patient perspective and be modified accordingly. There were also concerns about how much time and involvement are reasonable for patient partners.

#### 3.3.1 Patient perspective

Both groups described how integrating patient perspectives with traditional scientific research methods was at times challenging. Academic researchers described the tension between wanting to listen to, and consider patient partners' perspectives, while also making their own voices heard, without creating power imbalances. At some times, patient partner feedback created significant challenges for some academic researchers because it meant deviating from standard research practices and negotiating solutions that satisfied everyone.

Both groups also described how reconciling the relative rigidity of research with the patient engagement process could be difficult. Because research programmes are conducted within a complex interplay of existing scientific knowledge, ‘gold standard’ methods and priorities set by governments and funding bodies, academic researchers are sometimes limited in their ability to modify aspects of a programme. The development of patient‐oriented guidelines for administering the patient questionnaires is a good example of striking a balance between creating a more patient‐oriented tool without changing the instruments that are prevalidated standardized questionnaires (Table 3). This disagreement was resolved through team members' leadership, mutual respect, open‐mindedness and a strong desire to find a solution.

#### 3.3.2 Time commitment

Both groups recognized that the programme required heavy time commitment from patient partners. Some patient partners felt overwhelmed by the volume of material and data, while others appreciated having access to all of the documents and tools to fully understand the programme. Confronted with these opposing perspectives, academic research team members sometimes felt uncertain about the extent to which they could call for the involvement of patient partners.

Time constraints related to the research programme were also specifically highlighted by academic researchers, who sometimes felt that they did not have enough time to dedicate to patient engagement. For example, regarding the concerns surrounding the patient questionnaire (Table 3), patient partners expressed that their involvement in the preliminary steps of the data collection, such as in the identification and selection of the tools to be included in the patient questionnaire, could have saved time and sped up the development of the patient questionnaire.

### 3.4 Impact and value of patient engagement

Both groups recognized the added value of patient engagement, which brought new and diverse perspectives to the research programme. Patient partners' contributions were also highlighted in terms of improving the relevance of the research and the applicability of the results.

#### 3.4.1 Diverse perspectives and innovative ways of doing research

Both groups noted that the contributions of patient partners were diverse and could impact multiple stages of the research process, from programme planning to knowledge dissemination. Patient partners brought forth a patient perspective through their lived experience and skills and kept the research team focused on a patient‐centred approach. They encouraged flexibility and thoughtfulness around the approach and methods and acknowledged that a diversity of views enhances the research.

Academic researchers saw patient engagement as a novel way to take a step back and ‘see the bigger picture’, and to highlight the reasons for completing the work. Although academic researchers felt that patient engagement could sometimes be associated with delays, complications and tensions, the experience of working with a team of individuals with diverse skills and talents added value to the research. Academic researchers expressed that it was rewarding to be part of a movement that promotes patient engagement in research, leading some to encourage this approach among other teams.

#### 3.4.2 Improved research relevance and applicability

Patient engagement in PriCARE was seen as a unique opportunity to view the programme through a patient lens. Patient partners described this as providing the ‘critical patient/caregiver voice’. For academic researchers, this patient lens brought them closer to the frontline reality, which allowed them to identify research problems and solutions that were more important and relevant to patients. Similarly, patient partners felt that sharing their perspectives could ultimately improve the validity of research outcomes by identifying issues that would have otherwise remained blind spots for the research team.

Finally, through their feedback, patient partners contributed to the development of more accessible research tools, for example, the patient‐oriented guidelines for administering the patient questionnaires and the Patient Journey Map that visually depicts the patients' movement through the intervention and the various providers they encounter along the way. Academic researchers reflected on these contributions, noting how patient partners' lived experiences provided insight on how to better develop rapport with patients, thereby facilitating recruitment, participation rates and adherence to the intervention.

## 4 DISCUSSION

This study provides insight into how patient partners and academic researchers navigated the complex process of patient engagement in research and the challenges that accompany it. Perspectives of both groups provide a more accurate and complete picture of patient engagement in research and help emphasize aspects that are essential in promoting successful patient partner involvement. Overall, participants depicted patient engagement as an evolving and learning process where strategies to improve collaboration between patient partners and the academic research team are generated and developed over time, culminating in research that is ultimately stronger and more relevant, with the hope of improved outcomes for patients and a more efficient health care system.

Patient partners and academic researchers identified similar challenges and strategies related to team members' expectations, relationships, training, support and guidance. However, their experiences of patient engagement differed. They engaged in the programme with different backgrounds, motives and expectations. Patient partners experienced various levels of comfort over the course of the programme and expressed multiple strategies to minimize discomfort and maximize their involvement. Academic researchers voiced challenges related to successfully involving patient partners while ensuring the fidelity and validity of the research, and highlighted concerns regarding time constraints. A description of the challenges and strategies of patient engagement would not be complete without having access to both perspectives.

Previous studies on the barriers and facilitators of patient engagement in research stressed the importance of adequate planning and preparation of the academic research team members and patient partners.^9^, ^26^, ^39^ Providing clear role descriptions and expectations at the beginning of the programme, while staying flexible as the programme evolves, may promote team members' confidence about when and how to ‘do’ patient engagement, and how it fits into the research programme.^26^, ^40^ In addition, involving patient partners in the initial research stages may improve patients' comfort during data collection.^19^, ^25^, ^41^ Nevertheless, involving patients early in the process may increase the time required for patient engagement. Both academic research teams and funders must consider this additional time in the planning of research programmes. Finally, providing adequate and on‐going training to team members helps define their roles, promote positive attitudes towards patient engagement and foster trust and respect among team members, which may enhance the beneficial impact of patient engagement.^19^ Table 4 presents a summary of recommendations for patient engagement in research derived from each study theme.

**Table 4:** Summary of recommendations for patient engagement in research derived from each study theme.

Theme and description	Recommendations
Evolving relationships	Provide dedicated time and space for all team members to voice concerns or ideas related to the project. *For example: dedicate a specific period of time before or after team meetings for this*. Address team member concerns in a timely manner. Identify and plan concrete actions to address them and share them with the rest of the team. Encourage patient partner‐only meetings to promote peer‐to‐peer support. Actively encourage and seek out opinions of all team members whenever possible.
Creating an environment that fosters patient engagement	Recognize early on the additional time and resources required for patient engagement, and include those considerations when planning for the project and applying for research funding.
	*Roles and expectations*
	Clarify and codevelop roles of each team member early in the project and share this information with the rest of the team. Provide clear work expectations for each team member role. Periodically review roles and related expectations according to the project progress, and team member preferences and feedback.
	*Training*
	Actively inquire about patient partners' past experiences in patient engagement research and explore expectations for their roles in the current project. Facilitate access to trainings or resources related to the different roles of patient partners in the research process.
	*Compensation*
	Provide standardized compensation to all patient partners. Establish clear guidelines regarding patient partner compensation, including the list of activities to be compensated as part of the project. Communicate the proposed compensation to all patient partners before their integration in the research team.
Striking a balance	Include patient partners early on in the research process, including the study design, to facilitate participation in high‐impact decision‐making. Promote and encourage patient partner‐led initiatives to develop study tools. Recognize a mix of diverse experiences, perspectives and values.
Impact and value of patient engagement	Highlight patient partner contribution by recognizing small and big successes, providing compensation and including patient partners in multiple stages of the research process, from programme planning to knowledge dissemination. Assess, through qualitative or quantitative means, the impact of patient engagement in research.

Interestingly, while planning and early involvement of patient partners were deemed important, both groups also described patient engagement as a learning process requiring adaptations along the way. These seemingly contrasting ideas suggest the need for research teams to find a balance between providing a certain structure for patient engagement, while also remaining open to changing that structure. Flexibility in roles and team functioning may become increasingly important as team members discover their priorities, skills and limits, and as the group develops strategies to promote collaboration.^26^ Flexibility in defining and re‐defining patient partner roles may also help teams overcome challenges that arise along the way, such as personnel turnover or extended absences, which inevitably require some restructuring. In addition, this flexibility can be a means to support patient partner empowerment, as assigning roles too rigidly may result in a patient engagement process characterized solely by an exchange of services between patients and academic researchers.^40^ To be successful, the functioning of a team needs to build on a certain blueprint, which should then be regularly monitored and adapted along the way.

Other authors have previously commented on the need to provide a trusting and positive environment to foster patient engagement.^4^, ^15^ In a realist evaluation on patient and public involvement in 23 clinical research studies, Wilson et al.^42^ found that the relationship between academic researchers and patient partners was a key factor for successful patient engagement and that relationships took time to build. This highlights the need to allow time for a trusting relationship to develop and to adopt specific strategies to promote this, such as planning initial meetings to introduce each other, having dedicated time for informal exchange conversation before meetings and planning social events. In our study, while the participants highlighted the importance of having a positive and trusting environment, they also recognized that tensions could exist. In research with vulnerable populations, tensions and contradictions among the team members are largely inevitable because of possible differences in culture and inequity between the academic researchers and the patient partners.^43^

This is one of the few studies reporting experiences of patient engagement in research from the perspective of both groups, that is, the patient partners and the academic researchers. What is particularly novel in this study is that patient partners and researchers perceived challenges related to common themes, but with different lenses and experiences, which caused tensions between groups. These tensions, instead of paralysing the team and hindering the programme, often became opportunities for open discussions and new ways of doing. By taking the time to listen to the concerns of others, recognizing power relationships and negotiating ways to reach consensus, moments of tension constituted learning opportunities that could lead to improvements in the team's patient engagement approach.

## 5 LIMITATIONS

Since the experiences and challenges presented in this study are associated with a specific programme, all findings may not be transferable to other programmes involving patient partners, particularly if there are substantial differences in the nature of the research. Another limitation is that not all members of the PriCARE team were interviewed for the current study, therefore limiting the range of experiences presented. Moreover, participants are known to the research team and may feel compelled to focus on the positive engagement experiences and negate some of the negative experiences. The use of a participatory approach with patient partners and academic research team members acting as study participants and contributing to their own data analysis could potentially cause bias. Nevertheless, writing of this manuscript was led by a professional external to the research team (S. B.), which brought an external perspective. In addition, because both the academic research team and patient partners contributed to data analysis and manuscript preparation, this arguably promotes a more equal representation of experiences. Finally, the fact that the PriCARE research programme was conducted amid the COVID‐19 pandemic may have influenced patient partners' and academic researchers' experience in this programme. For example, the pandemic contributed to personnel turnover, and may have rendered communications and interactions more difficult among team members. Nevertheless, the team's success in conducting patient engagement in this unique context provides evidence that effective patient engagement teams can be built despite challenging situations.

## 6 CONCLUSION

Both patient partners and academic researchers highlighted the importance of finding a balance between providing a certain structure for patient engagement, while also remaining flexible to adaptations along the way. This study provides additional guidance to this end from the perspective of both groups.

## AUTHOR CONTRIBUTIONS

Catherine Hudon, Maud‐Christine Chouinard, Kris Aubrey‐Bassler, Fred Burge and Shelley Doucet contributed to the PriCARE research programme conception and design. Catherine Hudon, Mireille Lambert and Alya Danish led the different steps of the study. Alya Danish, Judy Porter, Donna Rubenstein and Mike Warren drafted the interview guide. Mathieu Bisson designed the analysis plan. Alannah Delahunty‐Pike, Olivier Dumont‐Samson, Dana Howse, Mireille Lambert and Cathy Scott analysed and interpreted the data with Judy Porter, Donna Rubenstein, Véronique Sabourin, Cathy Scott, Mike Warren and Linda Wilhelm. The first draft of the manuscript was written by Sophie Béland, Catherine Hudon and Mireille Lambert, and all authors commented on subsequent versions of the manuscript. All authors read and approved the final manuscript.

## CONFLICT OF INTEREST

The authors declare no conflict of interest.

## Data Availability

The data that support the findings of this study are available on request from the corresponding author. The data are not publicly available due to privacy or ethical restrictions.
